# Chest beats as an honest signal of body size in male mountain gorillas (*Gorilla beringei beringei*)

**DOI:** 10.1038/s41598-021-86261-8

**Published:** 2021-04-08

**Authors:** Edward Wright, Sven Grawunder, Eric Ndayishimiye, Jordi Galbany, Shannon C. McFarlin, Tara S. Stoinski, Martha M. Robbins

**Affiliations:** 1 Max Planck Institute for Evolutionary Anthropology, Deutscher Platz 6, 04103 Leipzig, Germany; 2 Goethe Universität Frankfurt Am Main, Frankfurt, Germany; 3 Dian Fossey Gorilla Fund International, Atlanta, GA USA; 4 Department of Anthropology, Center for the Advanced Study of Human Paleobiology, The George Washington University, Washington, DC USA; 5 Department of Clinical Psychology and Psychobiology, University of Barcelona, Barcelona, Spain

**Keywords:** Sexual selection, Zoology, Animal behaviour, Biological anthropology

## Abstract

Acoustic signals that reliably indicate body size, which usually determines competitive ability, are of particular interest for understanding how animals assess rivals and choose mates. Whereas body size tends to be negatively associated with formant dispersion in animal vocalizations, non-vocal signals have received little attention. Among the most emblematic sounds in the animal kingdom is the chest beat of gorillas, a non-vocal signal that is thought to be important in intra and inter-sexual competition, yet it is unclear whether it reliably indicates body size. We examined the relationship among body size (back breadth), peak frequency, and three temporal characteristics of the chest beat: duration, number of beats and beat rate from sound recordings of wild adult male mountain gorillas. Using linear mixed models, we found that larger males had significantly lower peak frequencies than smaller ones, but we found no consistent relationship between body size and the temporal characteristics measured. Taken together with earlier findings of positive correlations among male body size, dominance rank and reproductive success, we conclude that the gorilla chest beat is an honest signal of competitive ability. These results emphasize the potential of non-vocal signals to convey important information in mammal communication.

## Introduction

One of the many functions of animal communication is to mediate social interactions related to intrasexual competition and intersexual mate choice^1–3^. In particular, acoustic signals have been found to play a crucial role in facilitating assessment of rivals and in the choice of mates^1–4^. Acoustic signals can convey information about the sender’s competitive ability, body size and/or condition^5–7^. Signals are deemed honest when they provide reliable information about the sender^4^. Signals remain honest because they are directly linked to the sender’s physical characteristics, such as body size or condition that cannot be easily faked (called indexical signals), the costs of producing them are prohibitively high for low quality individuals (handicaps), or individuals who produce dishonest signals are punished and suffer reduced fitness^3,4,8,9^.

The relationship between body size and acoustic properties of signals are of particular interest in species in which body size determines fighting ability and reproductive success. Due to the allometric relationship between vocal tract length and body size, a strong association between the acoustic structure of vocalizations and body size has been observed^4,7^. For example, negative correlations between body size and formant dispersion (average spacing between resonant frequencies), indicating that (within-species) these are honest signals, have been found in a growing number of species such as rhesus macaques (*Macaca mulatta*)^10^, black and white colobus monkeys (*Colobus guereza*)^11^, red deer (*Cervus elaphus*)^5^, fallow deer (*Dama dama*)^12^, koalas (*Phascolarctos cinereus*)^13^, giant pandas (*Ailuropoda melanoleuca*)^14^, North American bison (*Bison bison*)^15^, southern elephant seals (*Mirounga leonine*)^16^, American alligators (*Alligator mississippiensis*)^17^ and corncrakes (*Crex crex*)^18^.

In contrast to vocal communication, the relationship between body size and non-vocal acoustic signals has received far less attention^19–21^. Non-vocal signals are also thought to be important in intrasexual competition and intersexual mate choice and thus under similar sexual selection pressure^1^. A study testing for the relationship between body size and acoustic structure of non-vocal signaling in mammals found that the peak frequency of knee clicking in eland (*Tragelaphus oryx*) reliably indicates body size^21^.

**Figure 1:**
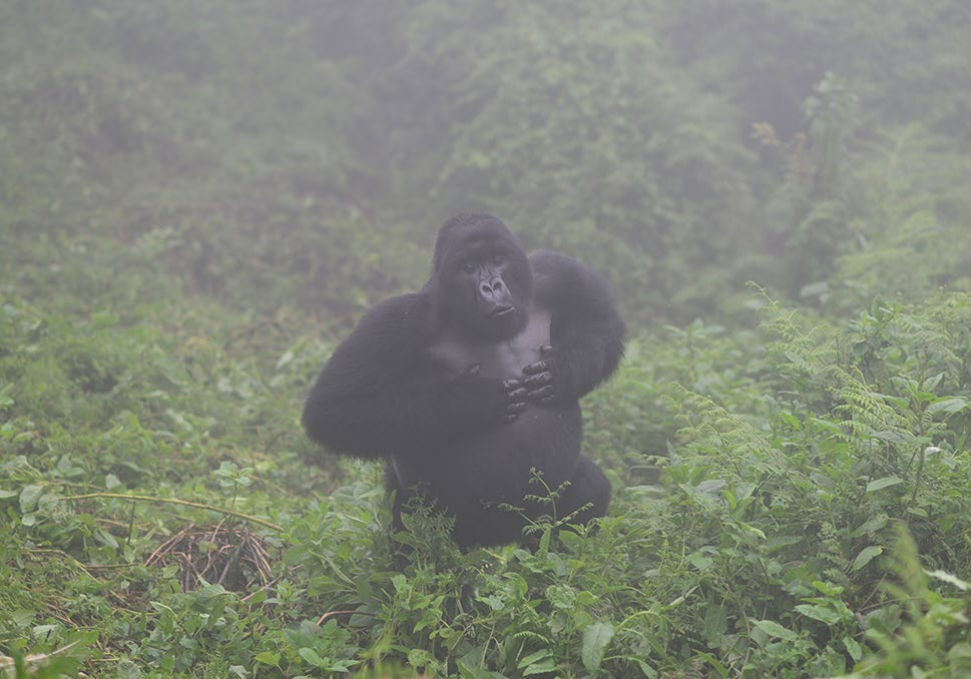
Mountain gorilla silverback PTO chest beating.

Another non-vocal signal is the chest beat which is the climax of the gorilla chest beating display^22^. Chest beating is performed when individuals usually rise bipedally and rapidly beat their chests with cupped hands in rapid succession, producing an impressive drumming sound (Fig. 1). The chest beat is sometimes preceded by a hooting vocalization and accompanied by slurred growling but not always^22–24^. The chest beat has both acoustic and visual components, and therefore is an example of a multimodal non-vocal signal. This multimodal long-distance signal, which can be heard over 1 km away, is most commonly performed by adult males (silverbacks)^22^. Gorillas are highly sexually dimorphic, living in predominantly one-male, multi-female social groups, resulting in high male–male competition^25,26^. Females may disperse among social groups, thereby exhibiting female choice for mates^27^. Previous research has suggested that the chest beat is an important signal in male–male competition and mate choice^22^. Silverbacks chest beat relatively infrequently, on average 0.5 times per 10 hours^23^, but may chest beat for as much as once every few minutes during intergroup encounters (M. Robbins personal observation). Silverbacks also chest beat more frequently on days when females are in estrous^28^. Body size of males is thought to reflect fighting ability as it strongly correlates with dominance rank in multi-male mountain gorilla groups and reproductive success in both species of gorillas^29–31^. However, it remains unclear what information chest beats convey and whether they reliably indicate the body size of the sender.

We examined whether the gorilla chest beat is an honest signal of body size. As a measure of body size we used back breadth, which is likely to be a good proxy for chest volume and therefore a relevant trait to correlate with acoustic properties of chest beats. First, we examined the relationship between body size and the peak frequency of the chest beats (the non-vocal drumming sound). We tested the prediction that larger males have significantly lower peak frequencies than smaller males, because there should be a direct relationship between the size of the animal producing the sound and low dominant frequency^32,33^. Second, we correlated body size to several temporal characteristics of chest beats, such as duration, number of beats and beat rate (number of beats per second). We tested the prediction that larger males chest beat for a longer duration, with a greater number of beats at a faster beat rate than smaller males. Because acoustic signaling is thought to be energetically costly to produce, larger males may be better equipped to produce them^34–37^.

## Results

### Frequency of chest beats

During 3211 h of focal animal sampling (mean per male = 128; range 8–307; *N* males = 25), we observed 503 chest beats, which is an average of 1.6 chest beats every ten observation hours (SD: 1.4; Table 1).

**Table 1:** Chest beats recorded during focal animal follows, together with back breadth and age of the mountain gorilla males (males in acoustic analysis are shown in bold).

Male	Social group	N chest beats	Focal time h	Chest beat rate per 10 h	Back breadth cm	Age year
**KRB**	PAB	40	90.1	4.4	59.8	16.5
**URS**	TIT	50	272.7	1.8	58.0	14.0
**GIC**	PAB	49	295.0	1.7	60.3	19.9
**PTO**	TIT	39	262.3	1.5	54.6	15.2
**DUS**	PAB	8	52.4	1.5	55.8	12.6
**AHZ**	PAB	1	11.1	0.9	58.7	12.1
IYA	IYA	50	114	4.4	64.6	14.3
MAF	MAF	51	125.8	4.1	62.2	15.8
GIR	GIR	23	57.0	4.0	62.1	20.5
ISH	PAB	27	90.7	3.0	61.5	13.8
IGZ	KUY	7	27.4	2.6	55.4	12.6
TBK	NTA	38	217.5	1.7	57.0	16.9
ISA	ISA	30	186.7	1.6	65.0	22.5
TRK	TIT	38	245.2	1.5	55.4	16.5
KBN	PAB	1	7.5	1.3	59.6	12.9
BWE	BWE	6	55.4	1.1	58.4	25.1
WAG	UGE	9	103.6	0.9	54.8	20.2
UGE	UGE	7	90.0	0.8	59.4	27.5
KBH	ISA	10	146.0	0.7	58.3	16.5
VUB	KUY	4	68.0	0.6	62.0	21.6
KIR	KUY	3	73.7	0.4	59.2	23.6
CAN	PAB	8	306.9	0.3	64.3	36.4
UGU	NTA	3	174.2	0.2	60.2	24.5
NTA	NTA	1	90.0	0.1	57.0	29.1
RAN	TIT	0	48.3	0.0	60.2	22.7
Total		503	3211.3			

Data for the number of chest beats were collected during focal animal follows to enable the calculation of a rate of chest beating. Due to the logistical difficulties of recording chest beats, we collected the acoustic recordings of chest beats at different times. Age is the mean age during the study period.

### Peak frequency

**Figure 2:**
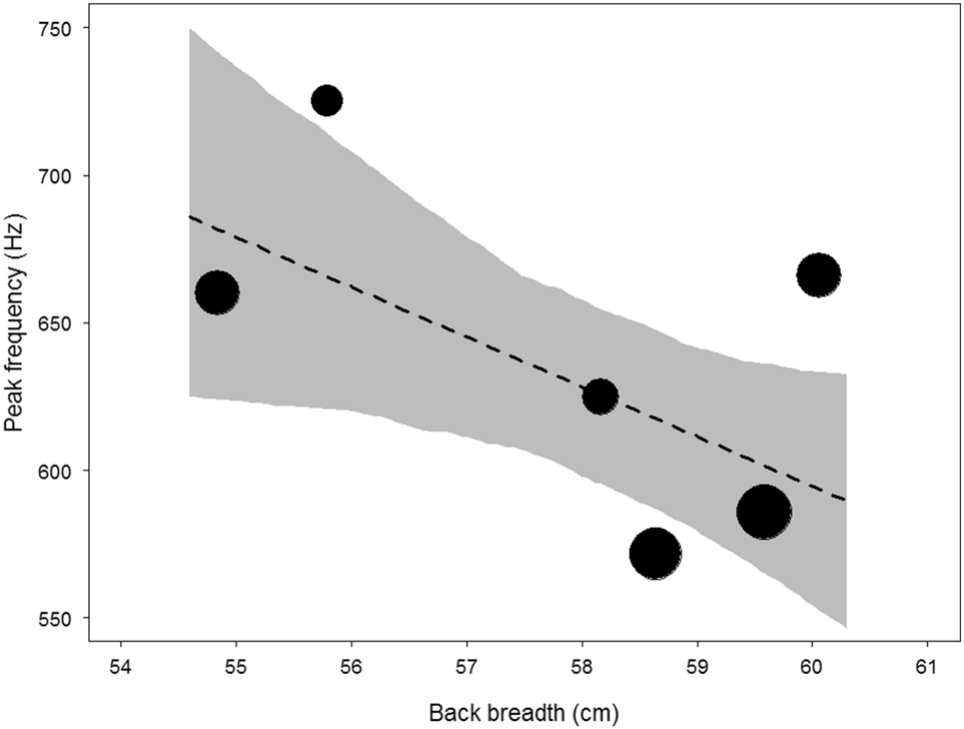
Relationship between body size (back breadth) and peak frequency of chest beats. The area of the circles represents sample size (*N* = 36). The dashed line is the fitted model after controlling for age and the shaded area is its 95% confidence intervals.

The mean peak frequency per male was 638.9 Hz (range: 459.0–1003.0 Hz; SD: 88.8; *N* data points = 36; *N* males = 6; Table 2). The mean within- and between-individual coefficients of variation (CV) for peak frequency were 13.3% and 15.5%, respectively. The chest beat of larger males comprised significantly lower peak frequencies than smaller males (Table 3; Fig. 2). An increase in one standard deviation in body size resulted in a decrease of 34.6 Hz in median peak frequency.

**Table 2:** Acoustic recordings and the variables examined. Acoustic recordings were made opportunistically at different observation times from the behavioural observations.

Male	Back breadth cm	N recordings	Peak Frequency Hz		Chest beat duration sec		Number of beats		Beat rate beats/sec	
			Mean	SD	Mean	SD	Mean	SD	Mean	SD
URS	58.0	4	624.75	35.05	0.30	0.09	4.25	0.50	14.45	2.10
GIC	60.3	6	665.83	22.60	0.48	0.11	7.17	1.94	14.86	1.24
KRB	59.8	9	585.56	48.91	0.45	0.16	5.89	2.15	13.16	2.16
PTO	54.6	6	660.17	90.22	0.46	0.17	6.33	1.75	14.07	1.80
DUS	55.8	3	725.33	272.18	1.77	0.98	18.67	8.02	11.09	1.41
AHZ	58.7	8	571.63	63.57	0.42	0.23	5.50	2.39	14.32	2.35
Mean			638.88	88.76	0.65	0.29	7.97	2.79	13.66	1.84

**Table 3:** The influence of body size (back breadth) and the control variable age on peak frequency of chest beats (Hz; *N* = 36).

Predictor	Estimate	SE	T value	CI _lower_	CI _upper_	*P*
Intercept	624.280	14.820	42.119	596.106	654.853	
Body size	− 34.570	16.260	− 2.127	− 63.987	− 2.871	0.048
Age	26.730	16.260	1.644	− 5.734	59.688	0.120

Body size and age were z-transformed to a mean of 0 and standard deviation of 1. Full null model comparison: χ^2^_1_ = 3.919; *P* = 0.048. CI: upper (97.5%) and lower (2.5%) confidence intervals.

### Chest beat duration

The mean chest beat duration per male was 0.65 s (range 0.17–2.82 s; SD: 0.29; *N* data points = 36; *N* males = 6; Table 2). The mean within- and between-individual CV for chest beat duration were 39.0% and 85.6%, respectively. Chest beat duration was not significantly associated with body size (full null model comparison χ^2^_1_ = 0.707; *P* = 0.401).

### Number of beats

The mean number of beats per chest beat and male was 8 (range 3–27; SD: 2.8; *N* data points = 36; *N* males = 6; Table 2). The mean within- and between-individual CV for the number of beats per chest beat were 31.6% and 67.9%, respectively. The number of beats per chest beat was not significantly associated with body size (full null model comparison χ^2^_1_ = 0.446; *P* = 0.504).

### Beat rate

The mean beat rate per chest beat and male was 13.7 beats/s (range 9.165–18.114; SD: 1.84; *N* data points = 36; *N* males = 6; Table 2). The mean within- and between-individual CV for beat rate were 13.5% and 10.1%, respectively. The beat rate was not significantly associated with body size (full null model comparison χ^2^_1_ = 0.125; *P* = 0.724).

In addition to back breadth we also repeated the above analyses using crest-back score, which is a composite measure combining back breadth and sagittal crest height^31^. The two body size measures are highly correlated^31^ so we did not fit a model that included both variables. The results of the models with only crest-back score were similar to the ones with back breadth, with crest-back score being significantly correlated with peak frequency, but not with any of the temporal measures (Supplementary Note and Table S1).

## Discussion

Our results indicate that mountain gorilla chest beats reliably convey information about the body size of the sender. Larger males consistently emitted chest beats with lower median peak frequencies than smaller males. This finding is an important contribution to the growing literature on honest signaling of body size in acoustic communication, which has predominantly focused on vocalizations^5,10–18^. This is one of a few studies in mammals demonstrating that body size is reliably encoded in a non-vocal acoustic signal. In eland bulls, body size (skeletal measures and muscle mass) was shown to be negatively correlated with the peak frequency of knee clicks^21^. In adult male gorillas, body size (a composite measure combining crest height and back breadth) is thought to reflect competitive ability because it correlates with dominance rank in multi-male groups and reproductive success^31^. Additionally, silverbacks chest beat more frequently on days when females are in estrous, presumably as a courtship display^28^. Taken together, this strongly suggests that the chest beat is an extremely important signal in intrasexual competition and intersexual mate choice in gorillas. Moreover, given that different forms of drumming behaviour, incorporating substrates other than the chest or body, are surprisingly common in a wide range of animals^19,20^, it is likely that this understudied non-vocal acoustic mode of communication functions to reliably indicate competitive ability in many other species as well.

Our measure of body size, back breadth, likely correlates with a range of morphological traits, including chest volume, pulmonary capacity and hand size. Therefore it is unclear which specific trait or traits are responsible for driving the inverse relationship between back breadth and peak frequency. Moreover, gorillas like other non-human primates possess laryngeal air sacs which are thought to act as resonators, enhancing acoustic signals^4,11,24^. Indeed, gorillas appear to use laryngeal air sacs during growling vocalizations which often accompany chest beating^24^. The volume of laryngeal air sacs is likely to be directly correlated with body size, at least within-species (which in orangutans (*Pongo*) can reach a massive 6 L^38^). Thus larger males are expected to have larger laryngeal air sacs than smaller males, further lowering the resonating non-vocal frequencies produced whilst chest beating. However, our knowledge of the size and function of laryngeal air sacs in primates and other taxa remains poor^39^.

Both dominant and subordinate male gorillas emitted chest beats. In general, gorilla males likely chest beat to attract estrous females and intimidate rivals^22,28,31^. However, younger subordinate males may also chest beat as a means to fine tune this signal and acquire social feedback from conspecifics. The importance of practice is evident as infants as young as one year of age commonly start emitting chest beats during social play^22,40^. Interestingly, the chest beat rate (number of chest beats per unit time) in the current study (2014–2016) was over three times higher than what was previously reported (1968–1969)^23^. We are unsure why this is the case, but it could be due to a number of different factors, including a higher number of estrous females per group, younger males, or more intergroup encounters over time^41^.

Even though we have demonstrated that chest beats reliably convey the body size of the emitter, future studies need to show that receivers actually attend to this information. Gorilla chest beats are thought to play a key role in male–male competition allowing individuals to assess the fighting ability of competitors and thus influence whether they should initiate, escalate or retreat in intra- and intergroup contests^22,31,42^. Similarly, male gelada baboons (*Theropithecus gelada*) assess the competitive ability of rivals through vocalizations and compare it to their own, governing how they respond in contests^43^. Intense contact aggression between males is infrequent in gorillas, which is presumed to reflect the high costs of aggression and their ability to resolve conflicts without resorting to this high risk behaviour (within-group^31,42^; between-group^44^). We expect that chest beats to also play a critical role in mate choice^22,28,45^, providing females with information about the size of the males in their own group and in neighbouring groups, which may influence their decision to transfer to another group. Larger alpha males lead groups with more adult females than smaller males, strongly suggesting that females actively chose to transfer into groups with large alpha males^29–31^. Gorillas may be similar to red deer hinds in their ability to discriminate between the acoustic signals of their current harem-holder stag and those of neighbouring stags^46^. Lastly, because chest beats can be heard over long-distances, we predict that both male size and the number of different males emitting chest beats are two important factors influencing group movement. Recent work in Bwindi mountain gorillas speculated that one of the functions of chest beats is to mediate how groups use space, with smaller groups with fewer adult males likely avoiding larger ones with more males, which would help to explain their findings that larger groups having more exclusive home ranges and core areas than smaller groups^47^.

We found no support for body size to influence the duration of chest beats, the number of beats, or the beat rate. Acoustic signals are thought to be energetically expensive to produce^34–37^ and we expected chest beats to be as well, with anecdotal accounts of gorillas that emit a high frequency of chest beats showing signs of exhaustion (personal observation). This is in contrast to studies of savannah baboons (*Papio ursinus*), showing that males with higher competitive ability produce longer vocalizations than weaker ones^48,49^. It is possible that the duration of chest beats (and the beat rate) decreases over time during periods of high chest beating frequency, and this decrease may be stronger in smaller males. In general the relationship between body size and the duration of vocalizations and other acoustic sounds has been understudied in mammals^4,50^.

In addition to conveying information on body size (and other phenotypic traits), we would expect it to be important for chest beats to be individual-specific, thereby allowing receivers to discriminate the identity of the emitter. Further study is needed to determine if there are individual signatures to the chest beats. Interestingly, we found smaller within-individual than between-individual coefficients of variation, particularly for chest beat duration and number of beats (39.0 vs. 85% and 31.6 vs. 67.9%, respectively). Notably, several temporal aspects of non-vocal drumming displays by chimpanzees (*Pan troglodytes*), ruffed grouse (*Bonasa umbellus*) and great spotted woodpeckers (*Dendrocopos major*) show significant individual variation, similar to many vocalizations in a wide range of species^19,51,52^. For example, the buttress drumming of individual chimpanzees significantly differ in the mean duration and the mean number of beats^52,53^.

The gorilla chest beat has both an acoustic and visual component, making it a multimodal signal. Individuals in visual proximity can benefit from seeing and hearing the gorilla emitting the chest beat, whereas individuals further away rely on the acoustic component. Researchers have been interested in determining whether the different components of multimodal signals convey the same (redundant signal or backup hypothesis) or different information (multiple messages hypotheses)^54^. Gorillas live in tropical forests with dense vegetation, meaning that it is often difficult to see conspecifics even if they are close by. Therefore, we argue that the evolution of the chest beat as a multimodal signal is at least in part to enhance signal transmission in an environment with limited visibility. We would expect the same messages to be transmitted in both visual and acoustic modalities, which would provide support for the redundant signal hypothesis. However, these two hypotheses are not mutually exclusive, as the chest beat signal may transmit additional information, other than body size, which is then repeated in the visual and acoustic modalities, providing support for both hypotheses.

## Methods

### Ethics statement

This observational study was done in accordance with guidelines of the Rwanda Development Board, Rwanda Ministry of Education, Dian Fossey Gorilla Fund and Max Planck Institute for Evolutionary Anthropology and adhered to all laws of Rwanda.

### Study population and behavioural data collection

The behavioural data collection was conducted on 25 wild, adult male silverback gorillas (older than 12 years) habituated to human observers, between January 2014 and July 2016, from ten social groups monitored by the Dian Fossey Gorilla Fund’s Karisoke Research Center, Volcanoes National Park, Rwanda (Table 1). We conducted focal animal sampling periods of approximately 50 min duration to record all agonistic behaviour (aggressive displays and any form of contact aggression), noting the identity of the male who initiated the interaction^31,55^. The focus here was on the aggressive displays involving a chest beat that were directed at conspecifics. These data were then used to calculate a chest beat rate per male.

### Sound recordings

We opportunistically collected sound recordings of silverback chest beats using a Sennheiser ME66 shotgun microphone and K6 power module with a MZW 66 windshield and a Zoom H4n recorder (Zoom corp., Tokyo, Japan). Chest beats were recorded at a sampling frequency of 96 kHz and sampling accuracy was 24-bit. Recording sensitivity was set at 80 for the duration of the study. Due to the logistically challenging nature of collecting sound recordings, we focused on six adult males from two social groups (PAB and TIT) between November 2015 and July 2016. We obtained a total of 36 chest beat sound recordings from six males (Table 2). In addition to the identity of the male that emitted the chest beat (sender), we also recorded the distance (using a distance meter; Leica Disto E7400x, Leica Geosystems AG, Heerbrugg, Switzerland) and the orientation of the sender with respect to the microphone to verify that these parameters did not influence peak frequency in any meaningful way. Neither the distance nor the orientation significantly correlated with peak frequency (distance: r_p_ = − 0.069; t = − 0.400; df = 33; p = 0.692; orientation: r_s_ = − 0.120; S = 7996.3; p = 0.493, respectively).

### Body size

We used the parallel laser method^56^ to measure back breadth, defined as the maximum distance across the shoulders, including the rounded portion of the arms (for details see Galbany et al.^57^; Wright et al.^31^; Table 1; in addition to back breadth we also repeated the analyses using crest-back score, a composite measure combining back breadth and crest height^31^—see Supplementary Note). Measurements were obtained from an average of ten photos (range 3—24) per individual, totaling 507 photos of 25 males.

### Acoustic analyses

**Figure 3:**
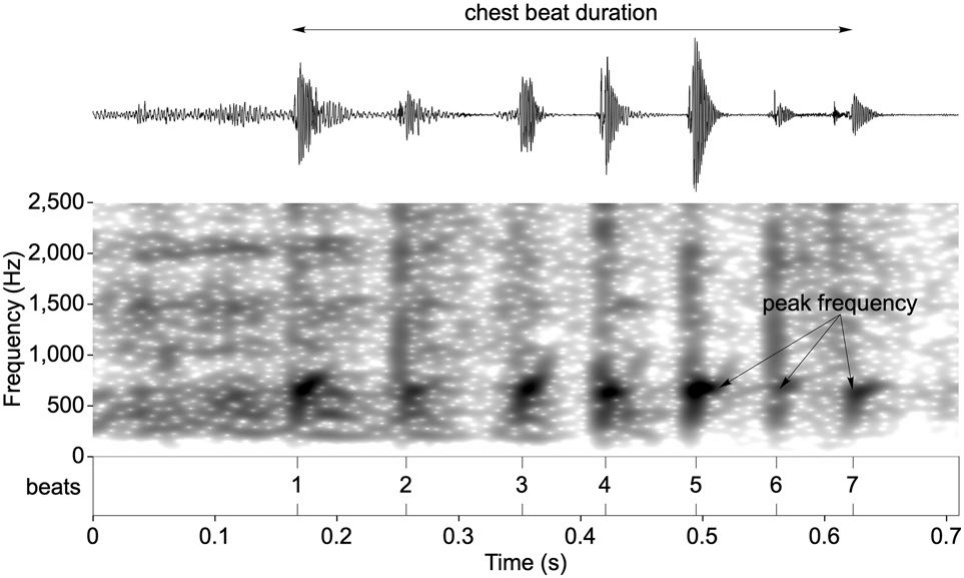
Spectrogram of a chest beat highlighting the response variables used in the analysis.

Acoustic analyses focused only on the non-vocal component of the gorilla chest beat display (drumming vibrations emanating from the beating of the chest). Recordings were down-sampled for analysis to 48 kHz sampling frequency and 16-bit sampling accuracy. Individual claps (beats) were identified as intensity spikes and marked in an annotation file (Fig. 3). Peak frequency was chosen as a single transparent measure to account for an observable frequency band of higher energy mainly between 500 and 1500 Hz^21^ (see Supplementary Methods and Fig. S1). The median peak frequency per chest beat was measured as frequency values of maximal intensity within the “fast Fourier transform spectrum” spectra over a 30 ms bandpass filtered window (50 to 2500 Hz) starting 5 ms prior to beat onset using the Praat software^58^.

### Statistical analyses

#### Peak frequency

To test the hypothesis of a significant negative correlation between body size and peak frequency we fitted a linear mixed effects model with Gaussian error and identity link. The response variable was the median peak frequency of each chest beat sequence (i.e., one final medianized data point per recording). The main predictor variable was back breadth. We included age as a control predictor and male identity as a random effect.

#### Duration, number of beats and beat rate

We then fitted three additional models examining the temporal characteristics of chest beats with the same structure with regard to the fixed effects, random effect and error structure as above but differing in the response variable. The first of these tested the prediction that larger males have significantly longer chest beat sequences than smaller ones. The response variable was therefore the duration of the chest beat (log-transformed). Next, we examined the prediction that larger males incorporated significantly more beats in their chest beats and have a faster beat rate than smaller males. In both these models the response variable was the number of beats during each chest beat. In the later model we also accounted for the duration of the chest beat sequence by including it as a control predictor.

The analyses were conducted in R (version 4.0.0)^59^ using the functions “lmer” of the “lme4” package^60^. Continuous predictors were z-transformed (to a mean of 0 and standard deviation of 1). We checked for normally distributed and homogenous residuals by visually inspecting qqplots and residuals plotted against fitted values. We verified that collinearity between age and body size was not an issue, by checking variance inflation factors (VIF) using the “vif” function from the “car” package (max VIF in all models was 1.2)^61^. We examined model stability by re-fitting the models after excluding levels from male identity one at a time, and comparing the estimates derived from these models with the estimates from the original model. No stability issues were found with regard to the main test predictor. The p-value for the main test predictor, body size, was computed using a likelihood ratio test comparing a full model with a reduced model not comprising this variable. Confidence intervals (95%) were determined using the function “bootMer” of the lme4 package^60^.

## Supplementary Information

Supplementary Information

## Data Availability

All data will be made fully available on reasonable request.
