# Supplementary material for: Chest beats as an honest signal of body size in male mountain gorillas (Gorilla beringei beringei)
Source: Sci Rep. 2021 Apr 8;11:6879. doi: 10.1038/s41598-021-86261-8 (PMC8032651; doi:10.1038/s41598-021-86261-8)
Supplement: Supplementary file 1 — Supplementary Information [file 41598_2021_86261_MOESM1_ESM.pdf]

Chest beats as an honest signal of body size in male mountain gorillas (*Gorilla beringei beringei*)

Edward Wright[1*], Sven Grawunder[1,2], Eric Ndayishimiye[3], Jordi Galbany[4,5], Shannon C. McFarlin[4], Tara S. Stoinski[3], Martha M. Robbins[1]

[1]Max Planck Institute for Evolutionary Anthropology, Deutscher Platz 6, 04103, Leipzig, Germany.

[2]Goethe Universität Frankfurt am Main, Germany.

[3]Dian Fossey Gorilla Fund International, Atlanta, Georgia, USA.

[4]Department of Anthropology, Center for the Advanced Study of Human Paleobiology, The George Washington University, Washington, District of Columbia, USA.

[5]Department of Clinical Psychology and Psychobiology, University of Barcelona, Barcelona, Spain.

[*]edward_wright@eva.mpg.de

Supplementary Information

Supplementary Note

We also fitted the models presented in the main text with crest-back score, a composite measure combining back breadth and crest height, instead of back breadth alone. Crest-back score was shown to positively correlate with dominance rank and reproductive success in male mountain gorillas[1]. The results with crest-back score were essentially the same as those with back breadth (Table S1).

Table S1. Full null model comparisons for the models presented in the main text fitted with crest-back score, instead of back breadth.

Model	χ^2	*P*
Peak frequency	4.223	0.040
Chest beat duration	0.167	0.683
Number of beats	0.096	0.757
Beat rate	0.019	0.891

The degrees of freedom for all models was 1.

Supplementary Methods

Peak frequency estimation was used by means of the PRAAT software[2]. Here for every data point the annotated chest beat was extracted 5 ms before and 25 ms after the beat onset. Beforehand the signal was bandpass-filtered (50 to 2500Hz) using a rectangular band filter in order to exclude influence of unrelated low- and high-frequency noise bands. The FFT spectrum (4441-bin FFT size) was then calculated and turned into a long term average spectra (LTAS) with a general bin width of 5.405 Hz (frequency resolution = 24000Hz/4441bins). The frequency step with maximal power spectral density (in dB) was then determined as *peak frequency* within the LTAS (Fig. S1).

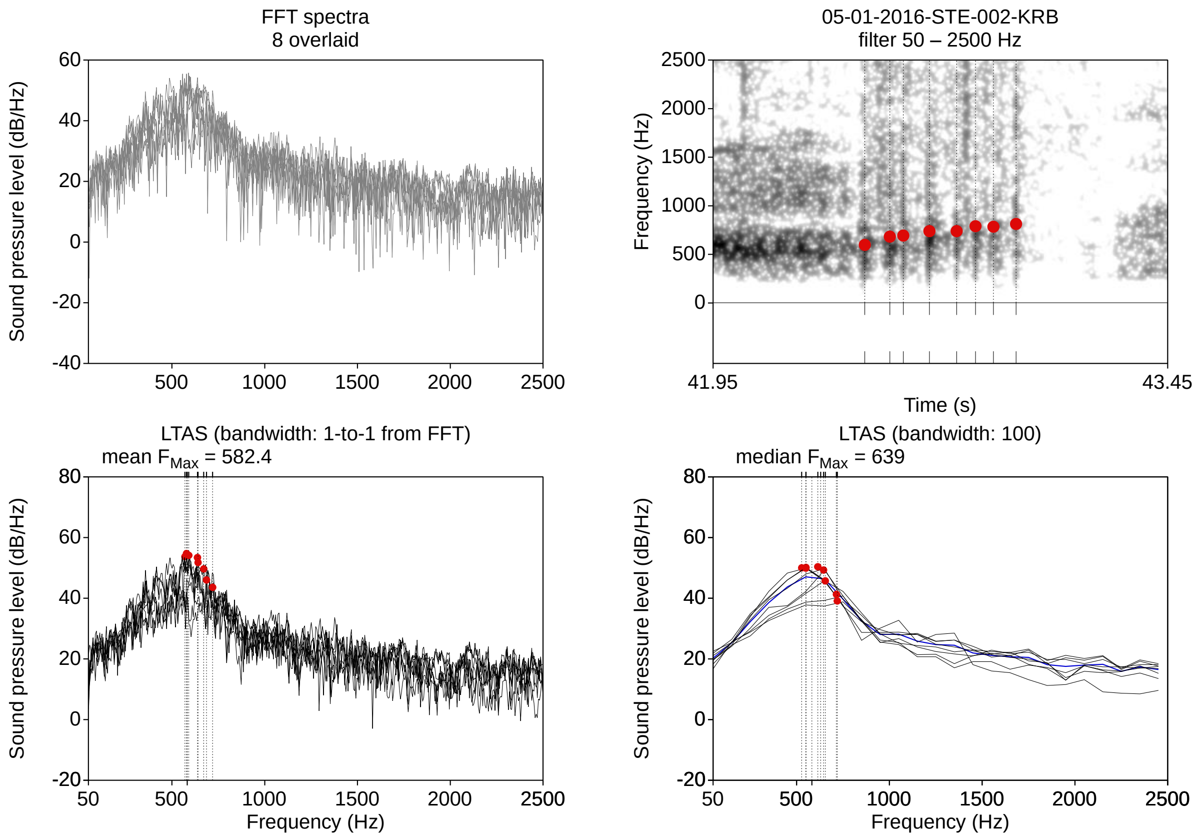

Figure S1. The two panels on the left side illustrate the FFT and derived LTAS spectra; red dots mark the positions of the peak frequencies; blue thick line (in lower right panel) corresponds to an averaged LTAS spectrum

References

1. Wright, E. *et al.* Male body size, dominance rank and strategic use of aggression in a group-living mammal. *Anim. Behav.* **151**, 87–102 (2019).
2. Boersma, P. & Weenink, D. Praat: doing phonetics by computer [Computer program] (2020). Version 6.1.36, retrieved 6 December 2020 from http://www.praat.org/.
